# Infection of Chinese Rhesus Monkeys with a Subtype C SHIV Resulted in Attenuated In Vivo Viral Replication Despite Successful Animal-to-Animal Serial Passages

**DOI:** 10.3390/v13030397

**Published:** 2021-03-02

**Authors:** Gerald K. Chege, Craig H. Adams, Alana T. Keyser, Valerie Bekker, Lynn Morris, Francois J. Villinger, Anna-Lise Williamson, Rosamund E. Chapman

**Affiliations:** 1 Primate Unit and Delft Animal Centre, Centre and Platform Office, South African Medical Research Council, Parow Valley, 7505 Cape Town, South Africa; 2 Division of Medical Virology, Department of Pathology, Institute of Infectious Disease and Molecular Medicine, University of Cape Town, Observatory, 7925 Cape Town, South Africa; Craig.Adams@uct.ac.za (C.H.A.); Alana.Keyser@uct.ac.za (A.T.K.); Anna-Lise.Williamson@uct.ac.za (A.-L.W.); Ros.Chapman@uct.ac.za (R.E.C.); 3 Centre for HIV and STIs, National Institute for Communicable Diseases, a Division of the National Health Laboratory Service, Sandringham, 2131 Johannesburg, South Africa; valerie.bekker@duke.edu (V.B.); Lynnm@nicd.ac.za (L.M.); 4 Antibody Immunity Research Unit, Faculty of Health Sciences, University of the Witwatersrand, Wits, 2050 Johannesburg, South Africa; 5 New Iberia Research Center, University of Louisiana, Louisiana, LA 70560, USA; francois.villinger@louisiana.edu

**Keywords:** SHIV, subtype C, Chinese rhesus macaques

## Abstract

Rhesus macaques can be readily infected with chimeric simian-human immunodeficiency viruses (SHIV) as a suitable virus challenge system for testing the efficacy of HIV vaccines. Three Chinese-origin rhesus macaques (ChRM) were inoculated intravenously (IV) with SHIVC109P4 in a rapid serial in vivo passage. SHIV recovered from the peripheral blood of the final ChRM was used to generate a ChRM-adapted virus challenge stock. This stock was titrated for the intrarectal route (IR) in 8 ChRMs using undiluted, 1:10 or 1:100 dilutions, to determine a suitable dose for use in future vaccine efficacy testing via repeated low-dose IR challenges. All 11 ChRMs were successfully infected, reaching similar median peak viraemias at 1–2 weeks post inoculation but undetectable levels by 8 weeks post inoculation. T-cell responses were detected in all animals and Tier 1 neutralizing antibodies (Nab) developed in 10 of 11 infected ChRMs. All ChRMs remained healthy and maintained normal CD4^+^ T cell counts. Sequence analyses showed >98% amino acid identity between the original inoculum and virus recovered at peak viraemia indicating only minimal changes in the env gene. Thus, while replication is limited over time, our adapted SHIV can be used to test for protection of virus acquisition in ChRMs.

## 1. Introduction

Despite the reported steady global decline of deaths caused by HIV, the number of new infections continues to be unacceptably high and the only cost-effective measure to curb this trend remains a protective vaccine. Initial HIV vaccine development relies on suitable animal models specifically designed to address select parts of the immune responses. Among these, nonhuman primate (NHP) models have gained prominence based on their close physiology and immune setup to man and the availability of lentiviruses that are not only able to infect NHPs but also cause pathology. Moreover, persistence of these viruses in NHP hosts even after prolonged antiretroviral treatments have led to a vigorous effort to investigate HIV cure in these models [1,2].

Infection of rhesus macaques (RM) with pathogenic chimeric viruses, simian-human immunodeficiency viruses (SHIVs) containing an increasing number of HIV-1 *env* genes, have been shown to induce simian AIDS [3], though less readily than the more virulent SIV models. However, SHIV have the distinct advantage to allow for testing vaccines and other prevention modalities that are specifically targeted to the HIV envelope, the first protein involved in transmission. This has led to salient advances in the design and testing of infection prevention in NHP models. Although Indian-origin RMs are by far the most well-established model of HIV/AIDS research, there is a global shortage of this subspecies [4,5], recently exacerbated by the COVID-19 pandemic. Chinese-origin RMs, on the other hand, are more readily available and can be infected with SIV or SHIV through various inoculation routes [5,6,7,8,9] as an alternative model [10]. Of note, the course of SIV and SHIV infection in Chinese RM is markedly slower compared with that of Indian RMs [6,7,9,11], suggesting that the kinetics of disease progression mimics human HIV/AIDS much more closely in Chinese RM and may therefore be a more relevant model for assessing the protective immune responses of candidate HIV vaccines.

In South Africa, HIV-1 clade C is responsible for nearly 95% of all HIV/AIDS cases [12,13], the vast majority of which are transmitted sexually by CCR5 (R5)-tropic viruses across mucosal surfaces [14]. For this reason, mucosally transmissible, pathogenic R5 SHIVs that encode HIV-1 clade C *env* genes are valuable as challenge viruses in the evaluation of protective responses of candidate HIV vaccines in NHP studies. Several SHIVs derived from clinical HIV-1 clade C isolates have been described [15,16,17,18,19,20,21,22,23] but only a few have been reported to be pathogenic in RMs after in vivo passage of their parental molecular clones [18,24,25]. Among the available clade C SHIVs, SHIVC109P4 [18] was of particular interest to us because it was derived from a southern African primary HIV-1 clade C isolate which had recently been transmitted heterosexually [26,27]. In addition, it has been classified as Tier 2 virus, similar to early founder HIV strains [28]. SHIVC109P4 has been adapted to in vivo replication and shown to be intra-rectally transmissible in Indian-origin RMs (InRMs) and to retain R5 tropism [18]. Furthermore, InRMs infected with the lineage-related SHIVC109P3 and SHIVC109P3N viruses progressed to AIDS and showed sex disparity in disease progression [25] as has been seen in humans infected with HIV. Overall, these studies demonstrated that these clade C SHIVs are pathogenic, replication-competent, R5-utilizing viruses with neutralization profiles similar to early founder HIV strains, indicating that they represent biologically relevant tools to evaluate the efficacy of candidate HIV vaccines in relevant NHP models.

In this study, SHIVC109P4 was adapted for replication in Chinese-origin RMs via a rapid 3three-step in vivo serial animal-to-animal passages via intravenous inoculations. A suitable dose for intra-rectal challenge of this Chinese-origin RM-adapted virus was determined for use in future vaccine efficacy testing. Plasma viral loads, SHIV-specific immune responses and CD4^+^ T cell counts were monitored and the CCR5 co-receptor usage confirmed.

## 2. Materials and Methods

### 2.1. Cell Lines and Culture Media

Mammalian cell lines for tropism: SupT1; REV CEM; Molt 4, clone 8, Molt 4 CCR5; CEMx 174 CEM, including the adherent cell line, U87 cells expressing the chemokine receptors, were provided by the NIH AIDS Research and Reference Reagents Program (ARRRP) (Germantown, MD, USA). HEK-293T cells and TZM-bl cells expressing CD4, CCR5 and CXCR4 and containing integrated reporter genes for firefly luciferase and galactosidase under the control of the HIV-1 long terminal repeat (LTR), were propagated in Dulbecco’s modified Eagle medium (DMEM)-l-Glutamax, supplemented with 10% foetal bovine serum (FBS) and penicillin/streptomycin (Pen-Strep; Gibco™, Invitrogen, Carlsbad, CA, USA). U87 cells, stably expressing CD4 and either CCR5 (U87CD4_CCR5) or CXCR4 (U87CD4_CXCR4), were maintained in DMEM supplemented with 10% FBS, Pen-Strep (Gibco™, Invitrogen, Carlsbad, CA, USA), 1 µg/mL puromycin (Sigma-Aldrich, St. Louis, MO, USA) and 300 µg/mL G418 (Geneticin; Invitrogen, Carlsbad, CA, USA). Non-adherent cell lines (SupT1; REV CEM; Molt 4 clone 8, Molt 4 CCR5; CEMx174) and rhesus peripheral blood mononuclear cells (PBMC) were cultured in RPMI-1640 medium containing 10% foetal calf serum, Pen-Strep, l-glutamine (R10 medium).

### 2.2. Expansion of SHIVC109P4 from Seed SHIV Stock

An aliquot of the original SHIVC109P4 stock [18] was expanded in vitro in Chinese-origin rhesus macaque (ChRM) PBMC to generate the initial inoculum stock (designated SHIVC109P4b). Rhesus PBMC were isolated from heparin-anticoagulated blood by standard ficoll-histopaque gradient centrifugation. The CD8^+^ cells in the PBMC isolated from normal uninfected ChRM blood were depleted in vitro using anti-CD8 monoclonal-coated magnetic beads (Dynabeads, Invitrogen, Carlsbad, CA, USA) according to the manufacturer’s guidelines and incubated in R10 medium containing concanavalin A (Con-A; 5 µg/mL) for 3 days. The PBMCs were then transferred into a 6-well plate (2 mL/well at 2–3 × 10^6^ cells/mL) and 500 µL/well of the untitered virus seed stock added. After 24 h in culture, the cells were transferred to a T_25_ cm^2^ flask and the total volume adjusted to 5 mL with R10 supplemented with 50 IU/mL recombinant rhesus macaque IL-2 (rIL-2; Courtesy of the Resource for nonhuman Primate Immune Reagents, Yerkes National Primate Research Center, Emory University, Atlanta, GA, USA). PBMC cultures were maintained at a cell density of 2–3 million/mL in R10 media containing 50 IU/mL of rIL-2. The culture supernatant was harvested every 3 to 4 days and replaced with an appropriate volume of R10 containing rIL-2 to maintain the cell density at 2 to 3 × 10^6^ cells/mL. Supernatants with >10 ng/mL SIV p27 were pooled to make a single virus stock.

### 2.3. SIV p27 ELISA and TCID_50_ Titre in TZM-bl Cells

Virus quantification in the culture supernatants was done by enzyme-linked immunosorbent assay (ELISA) for SIV p27 antigen using a commercial kit (Advanced Bioscience Laboratories, ABL, Rockville, MD, USA) following the manufacturer’s instructions. Supernatants with >10 ng/mL SIV p27 were pooled to make a single virus stock. The infectivity of the virus stock was assessed and the TCID_50_ was determined in TZM-bl cells. To do this, the virus stock was inoculated at 1:5 serial dilutions in quadruplicate wells of 96-well plates containing in TZM-bl cells in the presence of 20 µg/mL of DEAE-dextran hydrochloride (Sigma-Aldrich, St. Louis, MO, USA). Virus titre was determined 48 h post-infection by measuring the level of luciferase activity expressed in infected cells. The TCID_50_ was calculated as the dilution point at which 50% of the cultures were infected.

### 2.4. Animal Inoculations

**Figure 1:**
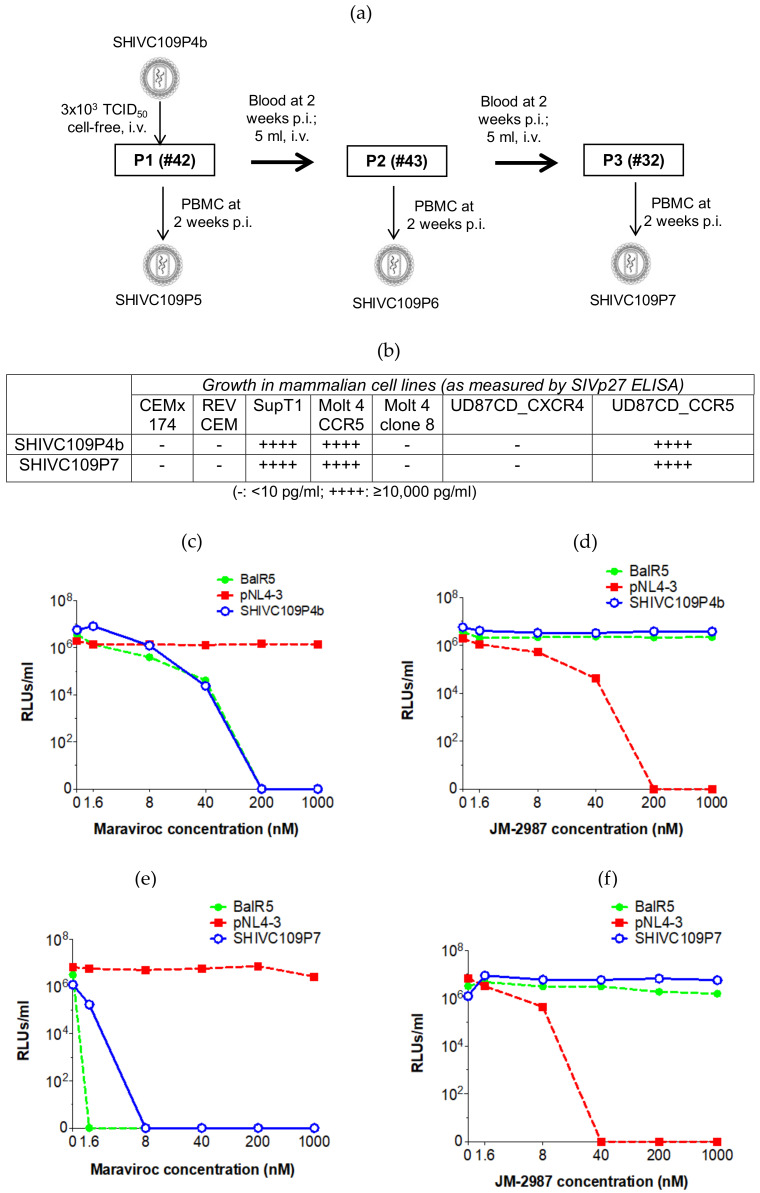
Generation and characterization of SHIV stocks (SHIVC109P4b and SHIVC109P7) which were derived from the parental virus, SHIVC109P4. (**a**) Schematic diagram showing how SHIVC109P4b was serially passaged in ChRMs to generate a macaque-adapted inoculum stock, SHIVC109P7. The cell tropism of SHIVC109P4 stocks, SHIVC109P4b and SHIVC109P7, for different mammalian cell lines (**b**) was assessed by measuring the level of viral replication in vitro using SIVp27 ELISA kits. In addition, the co-receptor usage of the SHIVC109P4b stock prior to passage in ChRMs (**c**,**d**) and SHIVC109P7 after passage (**e**,**f**) was determined using CCR5 (maraviroc) and CXCR4 (JM-2987) inhibitors. The effect of maraviroc (**c**,**e**)and JM-2987 (**d**,**f**)in a competitive inhibition assay to selectively block the entry of the viruses into TZM-bl cells was compared with control HIV-1 laboratory strains, BalR5 (R5-tropic) and pNL4-3 (X4-tropic). The line graphs show the level of viral replication of each virus in TZM-bl cells in the presence of serial dilutions of the co-receptor inhibitors. (IV: intravenous inoculation; PBMC: peripheral blood mononuclear cells isolated from heparinised whole blood).

Eleven ChRMs were used in this study and were randomly allocated to two groups, A (*n* = 3) and B (*n* = 8). All animals were seronegative for SIV, simian retrovirus type D (SRV), simian T-cell leukemia virus (STLV) and monkey B virus. Animals in group A were used for in vivo rapid animal-to-animal passages (as shown in Figure 1a) to generate a macaque-adapted SHIV inoculum stock. Briefly, the first animal (P1, animal #42) was inoculated intravenously (IV) with 3000 tissue culture infectious dose 50% (TCID_50_) of the initial inoculum (SHIVC109P4b) which was prepared from the original SHIVC109P4 viral stock. At 2 weeks post inoculation (p.i.), 20 ml of whole blood was collected from monkey P1 (animal #42) and 5 ml was transfused IV into macaque P2 (animal #43) while 15 ml were used to generate SHIVC109P5 virus stock as described below (Section 2.6). The SHIV replication was expected to peak at about 2 weeks p.i. and that was the rationale for choosing this time point for inoculation of the next animal for further virus adaption. At 2 weeks p.i., the same procedure was used to inoculated P3 (animal #32) with whole blood from P2 and to generate SHIVC109P6. At 2 weeks p.i. blood from the P3 (animal #32) was collected to generate the SHIVC109P7 inoculum stock for intra-rectal (IR) challenges in Group B animals. To determine a suitable dose for future IR challenge experiments, ChRMs in Group B were further divided into three subgroups, B1, B2 and B3 and inoculated weekly by IR route with different dilutions of the SHIVC109P7 virus stock until they became infected. Animals in Subgroup B1 (#35 and #36), were inoculated with 2 ml of undiluted SHIVC109P7, animals in Subgroup B2 (#37 and #38), were inoculated with a 1:10 dilution and animals in Subgroup B3 (#47, #48, #51 and #53) were inoculated with a 1:100 dilution of SHIVC109P7. The animal protocol was independently reviewed and approved by the Faculty of Health Sciences Animal Ethics Committee of the University of Cape Town and the Ethics Committee for Research in Animals of the South African Medical Research Council where the experimental animals were housed. The housing of animals was in line with the guidelines of the South African National Standards (SANS) on the Use of Animals for Research Purposes (SANS Code: 2008). Peripheral blood was obtained by venipuncture of the femoral vein and aseptically collected into vacutainers blood tubes containing heparin or EDTA at various time points pre- and post- inoculation. All animal procedures were performed by personnel who were authorized or registered by the South African Veterinary Council.

### 2.5. Processing of Blood Sample

Whole heparin anticoagulated blood was used for separation of plasma and isolation of PBMC by Ficoll-Histopaque gradient centrifugation. The PBMC were used for interferon-γ (IFN-γ) T cell ELISpot assays and in vitro tissue culture while the plasma was used for virus neutralization assays. Plasma and buffy coats were separated from whole EDTA anticoagulated blood for measurements of plasma viral RNA and proviral DNA, respectively, while some aliquots of whole EDTA blood was used directly for absolute CD4^+^ T cell counts.

### 2.6. Virus Isolation from the Blood of SHIV-Infected Animals and the Generation of ChRM-Adapted SHIV Stock

PBMC isolated from peripheral blood obtained from SHIV-infected ChRM (through IV in vivo passage) at 2 weeks p.i. were co-cultured with Con-A-stimulated, CD8^+^ cell-depleted, donor PBMC isolated from normal uninfected ChRM blood. Two approaches were used for the co-culture. In the first approach, unstimulated PBMC from the SHIV-infected animals were directly co-cultured with the Con-A-stimulated, CD8^+^ cell-depleted donor PBMC while in the second approach, the PBMC from the SHIV-infected animals were first stimulated for 3 days with Con-A (5 µg/mL) and subsequently cocultured with the Con-A-stimulated, CD8^+^ cell-depleted donor PBMC. The co-cultures were maintained in R10 containing 50 IU/mL rIL-2 for 21 days. To generate a ChRM-adapted SHIV stock, SHIVC109P7 that was used for IR inoculations, the culture supernatants harvested at days 9 and 13 from the P3 animal (#32) using the unstimulated PBMC approach, both of which had with >10 ng/mL SIV p27 were pooled and further expanded in ChRM donor PBMC as described for the SHIVC109P4b above (Section 2.2).

### 2.7. Tropism and Co-Receptor Usage

The co-receptor inhibitors, JM-2987 and maraviroc, were obtained from the ARRRP and were diluted as per manufacturer’s instructions. TZM-bl cells were plated at 2 × 10^4^ cells/well in 96-well flat-bottom plates. TZM-bl cells were incubated for 1 h in culture medium containing 5-fold dilutions of the inhibitors ranging from 1.6 to 1000 nM. After 1 h, 200 TCID_50_ of SHIV stock (SHIVC109P4b) or control HIV-1 isolates (pNL4-3 or BalR5) were added to the wells and 48 h later the cells were lysed and analyzed using Bright*-Glo™* Luciferase Assay System (Promega, Madison, WI, USA) with luminescence measured on a Glomax 96 microplate luminometer (Promega, Madison, WI, USA). Phenotypic tropism assays [29,30] were performed using permissive mammalian cell lines (SupT1; REV CEM; Molt 4, clone 8, Molt 4 CCR5; CEMx174 and UD87 cells), expressing CXCR4 (X4) and/or CCR5 (R5) receptors. After 5 days of SHIV inoculation, cell culture supernatant was collected for viral content determination using SIV p27 ELISA.

### 2.8. Measurement of Plasma Viral RNA Levels

Plasma viral RNA was isolated using QiaAmp viral RNA minikit (Qiagen, Valencia, CA, USA) and viral RNA levels were measured by quantitative reverse-transcriptase PCR (qRT-PCR) for SIV gag sequences, as described elsewhere [31]. A standard curve, with a 100-fold, diluted range of 10^0^ to 10^8^ gag copies, was generated using a SIV gag RNA stock (donated by Yerkes National Primate Research Center, Emory University, Atlanta, GA, USA). Sampling was done at pre-inoculation and various times p.i. The limit of detection was estimated at 16 RNA copy per mL.

### 2.9. Measurement of Proviral DNA Levels in the Blood

Two millilitres of EDTA anticoagulated whole blood was centrifuged and the whole layer of the white blood cells (200 µL buffy coat) was harvested. Genomic DNA was extracted from the 200 µL buffy coat using the QIAamp blood kit (Qiagen, Valencia, CA, USA). The qRT-PCR primers and probe amplified a 310-bp fragment of the SIV gag gene. The standard curve was generated by a 5-fold serial dilution from 50,000 copies of plasmid DNA down to 3.2 copies. qRT-PCR was done on a QuantStudio™ 7 Flex Real-Time PCR System (Applied Biosystems, ThermoFisher Scientific, Waltham, MA, USA), using TaqMan probe chemistry (TaqMan™ Gene Expression Master Mix; ThermoFisher Scientific, Waltham, MA, USA), as described elsewhere [31]. Data were expressed as the number of SIV DNA copies per ml of whole blood.

### 2.10. Measurement of Viral RNA and Proviral DNA Levels in the Tissues

RNA and genomic DNA was extracted from ten million cryo-preserved splenocytes and mononuclear cells from the tissues obtained at necropsy using the QIAamp blood kit (Qiagen, Valencia, CA, USA). The viral RNA and proviral DNA levels were measured by quantitative reverse-transcriptase PCR (qRT-PCR) for SIV gag sequences, as described above (Section 2.8 and Section 2.9). Data were expressed as the number of SIV gag RNA or DNA copies per 10 million cells.

### 2.11. Measurement of Absolute CD4^+^ T Cells

Whole EDTA-anticoagulated blood obtained at pre-inoculation and various time points p.i. from animals in Group B was used to determine the CD4^+^ T cell counts in the peripheral blood using the reagents from BD Biosciences (Franklin Lakes, NJ, USA) according to the manufacturer’s guidelines. An antibody cocktail comprising of 5 µL of each of the following: CD3 FITC (clone SP-34), CD4 PE (clone L200), CD45 PerCP (clone D058-1283) was added into each BD Trucount™ tube. Data was acquired on the BD FACSCalibur using the BD Multiset software (BD Biosciences, Franklin Lakes, NJ, USA) and analyzed using FlowJo 9 (TreeStar, LaJolla, CA, USA). The absolute CD4 count was calculated by dividing the number of positive CD4^+^ T cell events by the number of bead events and then multiplying by the BD Trucount bead concentration. The results are shown as the number of CD4^+^ T cells per µL blood.

### 2.12. IFN-γ ELISpot Assay

The assay was performed as previously described [32]. The PBMCs were incubated at 37 °C for 22–24 h in triplicates in the presence or absence of SIV Gag (1 µg/mL) or HIV-1 Env (1 µg/mL) peptide pools and PHA-M (10 µg/mL) as positive control. The spot counts were normalized to spot forming units (SFU) per million PBMC after subtracting the background (in absence of peptide pools). Readings below the cut-off value (30 SFU/million PBMC) were interpreted as negative responses and assigned a magnitude of zero. The results are given as cumulative SFU/million PBMC for both peptide pools.

### 2.13. Virus Neutralization Assay

Antibody neutralizing activity in the plasma obtained from monkeys at pre-inoculation and various time points p.i. was measured against subtype C HIV pseudoviruses as previously described [33]. Briefly, HIV Env-pseudotyped viruses from a Tier 2 global panel (Tro.11, CNE8, CNE55, 398F1_F6_20, HIV-25710-2-43, X2278_C2_B6, BJOX002000.03.2, CE1176_A3, X1632_S2_B10, 246F3_C10_2, CH119.10 and CEO217) and Tier 1A pseudovirus (MW965.26) that were previously titrated for optimal infectivity were added to serial dilutions of monkey sera or plasma in 96-well culture plates and freshly trypsinized TZM-bl cells were added. The plates were incubated at 37 °C for 48 h and neutralization activity measured as a function of reduction in Tat-regulated luciferase report gene expression in TZM-bl cells. Titres were calculated as the reciprocal of the highest plasma dilution resulting in a 50% reduction in relative luciferase units [RLU]. A titre <40 was judged negative for neutralization.

### 2.14. Full-Length Envelope Sequencing

For sequence analysis, viral RNA was prepared from plasma by using a RNA extraction kit (Qiagen, Valencia, CA, USA), followed by reverse transcription with SuperScript III reverse transcriptase (Invitrogen) and random hexamer primers (Amersham Pharmacia, Piscataway, NJ, USA). For the single genome amplification (SGAs), cDNA was titrated through nested-PCR of HIV-1 *env* of 9 replicates/dilution. The dilution that provided 2/9 (less than 30%) positive reactions was used to generate HIV-1 *env* single genome amplicons [34,35]. Sanger sequencing of HIV-1 *env* single genome amplicons, as previously described [36], was performed by the Central Analytical Facility at Stellenbosch University. Contigs of Sanger reads were assembled with CLC Sequence Viewer 6.7.1 software (CLC bio A/S, Aarhus, Denmark). At least 10 independent contigs per time-point were assembled.

## 3. Results

In this study, SHIVC109P4 was adapted for replication in ChRMs via 3 rapid in vivo serial animal-to-animal passages (Figure 1a). An aliquot of the original SHIVC109P4 stock [18] was initially expanded in ChRM PBMC to generate the inoculum stock (SHIVC109P4b). An aliquot from this stock was then used for IV inoculation of the first animal (P1, animal #42). Two weeks p.i., 5 mL of heparinised whole blood collected from monkey P1 was transfused directly to the second animal (P2, animal #43). Similarly, 5mL of blood from P2 collected at 2 weeks p.i. was transfused IV to the third monkey (P3, animal #32). In addition, PBMC were isolated from the blood collected from each animal at 2 weeks p.i. for virus isolation as described in the Methods section. The virus recovered from P3 (animal #32) representing the 3rd in vivo animal-to-animal passage (Figure 1a) was used to generate the inoculum stock (SHIVC109P7), which was then used for in vivo titration in Group B animals by IR inoculation.

### 3.1. Characterization of SHIV Inoculum Stocks

As described in the Materials and Methods section, two SHIV inoculum stocks, SHIVC109P4b and SHIVC109P7, were generated from the parental SHIVC109P4 seed stock. SHIVC109P4b was prepared by expanding the parental seeding stock in ChRM PBMC while the SHIVC109P7 was the recovered virus from P3 passage (animal #32) after the 3-step passage. Both SHIVC109P4b and SHIVC109P7 were titrated and characterized for tropism to common human cell lines and coreceptor usage. Both SHIVC109P4b and SHIVC109P7 replicated to high titres (SIV gag >1000 pg/mL) in R5 coreceptor-expressing cell lines (SupT1, Molt4 CCR5 and UD87_CCR5) but not in those expressing the X4 and lacking the R5 co-receptor (Figure 1b). Rev-CEM expresses CD4, CXCR4 and only very low levels of CCR5 [37], while CEMx174 does not express CCR5 receptors [38]. Therefore, these cell lines did not support virus entry. The exclusive use of R5 co-receptor was further confirmed by comparing the replication of SHIVC109P4b and SHIVC109P7 with HIV-1 strains that are known to exclusively use X4 (pNL4-3) or R5 (BalR5) in TZM-bl cells in the presence or absence of serial dilutions of X4 (JM2987) or R5 (maraviroc) inhibitors. TZM-bl cells express both X4 and R5 co-receptors and these inhibitors compete with the viruses for binding to the co-receptors thus limiting viral entry into the cells. As shown in Figure 1c,d, like the R5-tropic virus BalR5, the replication of SHIVC109P4b and SHIVC109P7 in TZM-bl cells was blocked by maraviroc in a concentration-dependent manner whilst X4 blockade by JM2987 had no effect. The levels of replication were inversely proportional to the concentration of the inhibitors for the respective sensitive viruses. This data showed that SHIVC109P4 retained the exclusive utilization of the CCR5 co-receptor after the serial passages.

### 3.2. Virus Isolation from the Peripheral Blood of IV-Infected Animals from Passages 1, 2 and 3

At 2 weeks p.i., PBMC isolated from P1 (animal #42), P2 (animal #43) and P3 (animal #32) were used for virus isolation using two differing approaches of in vitro co-culture method as described in Materials and Methods. Both methods were successful in recovery of virus from the PBMC. However, the approach involving co-cultivation of unstimulated PBMC from infected animals with Con-A activated, CD8-depleted PBMC from uninfected donor monkey was more robust in viral yield, in general, than the approach utilizing Con-A stimulated PBMC from the infected PBMC (Supplementary Figure S1). This may be due in part to the suppression of viral replication by the activation of CD8 T cells from the infected monkeys.

### 3.3. Plasma Viral RNA Loads of IV-Infected Animals from Passages 1, 2 and 3

**Figure 2:**
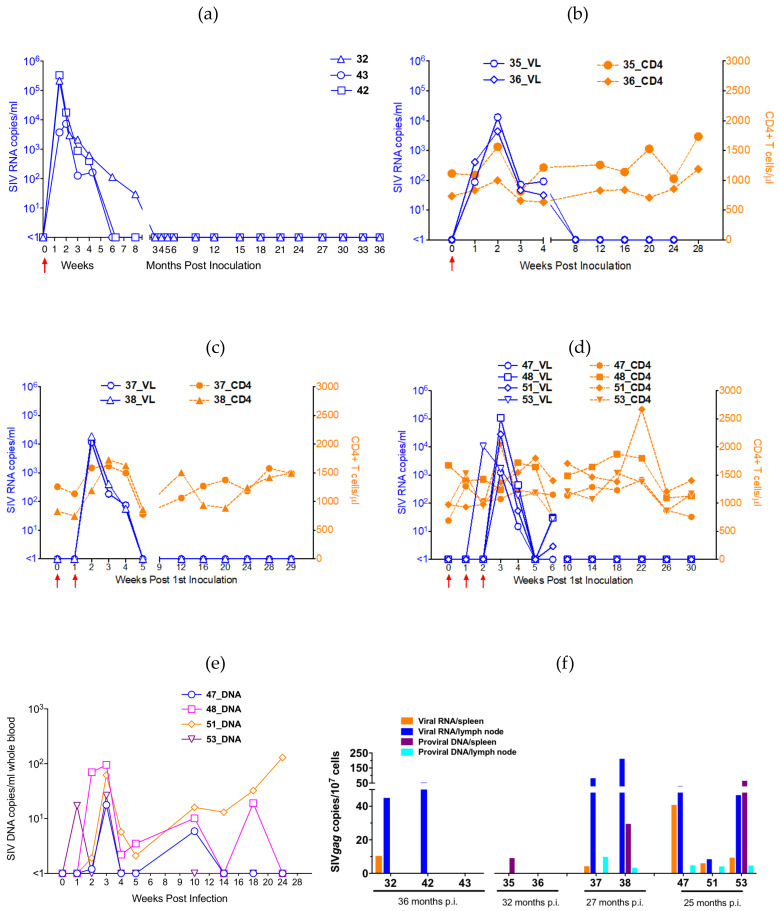
Plasma viral loads and CD4^+^ T cell counts pre-inoculation and at various time points p.i. (**a**) Shows the viral loads in the peripheral blood of monkeys 42, 43 and 32 which were inoculated IV with SHIVC109P4b, SHIVC109P5 and SHIVC109P6 respectively. SHIVC109P4b was prepared from the original SHIVC109P4 by in vitro expansion. Plasma viral loads (solid blue lines & symbols) and absolute CD4^+^ T cell counts (dashed orange lines & symbols) in the peripheral blood of ChRMs inoculated IR with 1:1 dilution (**b**), 1:10 dilution (**c**) and 1:100 dilution (**d**) of SHIVC109P7. Viral loads are also depicted as levels of SIV proviral DNA in the blood of ChRMs inoculated IR with 1:100 dilution of SHIVC109P7 at various time points (**e**) and both as SIV viral RNA and proviral DNA in the tissues (splenocytes and mononuclear cells extracted from either inguinal or axillary lymph node) at necropsy at the times indicated (**f**). Each line graph (**a** to **e**) represents an individual animal at pre-inoculation and p.i. with SHIV and each bar (**f**) represents a single tissue, either spleen or inguinal or axillary lymph node.↑: indicates inoculation times.

We monitored plasma viral RNA copies in the peripheral blood of the passaged animals (Group A) for 3 years p.i. As expected, viraemia was detected within the first week after IV inoculation, reaching a median peak of 2.15 × 10^4^ viral RNA/mL (range: 7.34 × 10^3^ to 3.29 × 10^5^) at 1 or 2 weeks p.i. (Figure 2a). The viraemia in these animals declined quickly, to undetectable levels by 6 (P1 and P2 animals) or 12 (P3 animal) weeks p.i. The viral loads subsequently remained undetectable throughout the experimental period of 36 months in all animals.

### 3.4. Plasma Viral RNA Loads and Absolute CD4^+^ T Cell Counts of Animals Infected IR with Different Dilutions of SHIVC109P7

In Group B animals, we monitored both the plasma viral RNA loads and the absolute CD4^+^ T cell counts for 28 weeks. The viraemia in those receiving undiluted (a 1:1 dilution of the) SHIVC109P7 virus stock (Subgroup B1) was detectable within the first week of inoculation like the IV-infected group with similar median peak viraemia (8.73 × 10^3^ viral RNA/ml; range: 4.4 × 10^3^ to 1.31 × 10^4^) (Figure 2b). Viraemia in these animals fell below the detection level by 8 weeks p.i. For animals receiving a 1:10 dilution of the virus stock (Subgroup B2), the animals became infected after two IR challenges, with viraemia reaching the peak within the first week of detection (median: 1.52 × 10^4^ viral RNA/mL; range: 1.12 × 10^4^ to 1.88 × 0^4^) p.i. (Figure 2c). Viraemia in this group became undetectable by 4 weeks p.i. For the final IR-infected group (Subgroup B3), 3 of the 4 animals required three IR challenges with the 1:100 dilution of SHIVC109P7 stock to become infected and 1 was successfully infected after the second challenge. Peak viraemia was reached within the first week of successful infection, with the median peak being 1.93 × 10^4^ viral RNA/mL (range: 1.25 × 10^3^ to 1.09 × 10^5^) (Figure 2d). Similar to many other SHIV-infected animals, the plasma viraemia declined quickly to undetectable levels between 3 and 9 weeks after initial detection. In all animals, the viral loads subsequently remained below the detection levels throughout the experimental period. Overall, both IV- and IR- infected groups had similar median peak viral loads at 2.15 × 10^5^ for the IV-infected (range: 7.34 × 10^3^ to 3.29 × 10^5^) and 1.23 × 10^4^ (range: 1.25 × 10^3^ to 1.09 × 10^5^) for the IR-infected animals put together (Subgroups B1, B2 and B3). There was no statistical difference in the median peak viral loads between the IV- and IR- infected groups (*p* = 0.278; Mann-Whitney test) or between the subgroups of IR-infected animals (*p* = 0.652; Kruskal-Wallis test). The median pre-infection CD4^+^ T cell counts in IR-infected animals was 1061/µL blood (range: 691 to 1673) (Figure 2b–d). The variations in median CD4 counts were modest after SHIV infection was confirmed and ranged from 1007 to 1417 cells per µL blood at various time points p.i. which was within the expected normal range of 500–2500 cells per µl blood.

### 3.5. Viral RNA and Proviral DNA Loads in the Blood and Tissues

We measured the level of SHIV provirus in the peripheral blood of Subgroup B3 animals (IR-infected with 1:100 SHIVC109P7 stock) pre-infection and at selected time points p.i. as SIV *gag* DNA copies. As shown in Figure 2e, proviral DNA levels peaked at 3 weeks p.i. for the majority of animals (3 of 4) ranging from 17 to 95 copies/ml of whole blood while the number of copies for animal #51 increased with time after the initial peak at week 3 p.i. to 130 copies/ml at week 24. For the majority of animals (3 of 4) fluctuating levels of the provirus were detected after week 8 when the virus was undetectable in plasma indicating persistent infection.

We also measured the levels of both viral RNA and proviral DNA in cryo-preserved cells that were extracted from selected tissues obtained at necropsy. Splenocytes and mononuclear cells from one of the peripheral lymph nodes (either inguinal or axillary lymph node depending on availability) were analysed from all the IV- and 7 of 8 IR-inoculated animals (tissues from monkey #48 were unavailable). As shown in Figure 2f, viral RNA was detected in the lymph nodes of 7 of the 10 animals and in the spleens of 5 of the 10 animals. Proviral DNA was measured in the lymph nodes and spleens of 5 and 3 animals respectively. Overall, varying levels of SHIV and SHIV provirus was detected in 8 of 10 animals in both IV- and IR- inoculated groups confirming the presence of a latent viral reservoirs in these animals, up to 36 months p.i. SHIV was undetectable in only 2 animals.

### 3.6. IFN-γ ELISpot Responses

**Figure 3:**
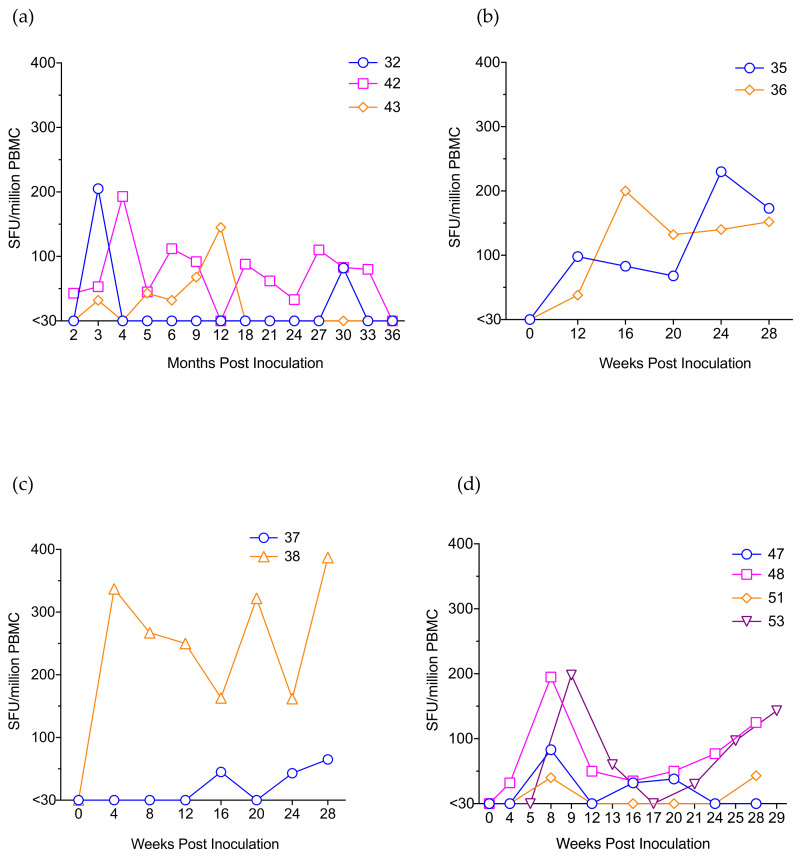
IFN-γ ELISpot responses in ChRMs following infection. (**a**) ChRMs (#32, #42, #43) inoculated IV with the passaged viruses. ChRMs inoculated IR with 1:1 dilution (#35, #36) (**b**), 1:10 dilution (#37, #38) (**c**) and 1:100 dilution (#47, #48, #51, #53) (**d**) of SHIVC109P7. The line graphs represent cumulative ELISpot responses to SIV Gag and HIV-1 Env peptide pools presented as spot-forming units (SFU) per million PBMC at each time point. Individual responses below the cut-off value of 30 SFU per million PBMC were considered negative and assigned a zero value.

SHIV-specific IFN-γ ELISpot responses were measured pre-inoculation and at various time points p.i. starting at weeks 4 (Groups B2 & B3), 8 (Group A) or 12 (Group B1). Responses were detected as early as week 4 for a few animals (2 of 6) and by 12 weeks in all except one animal (#37) which showed its first response at week 16 p.i. (Figure 3). Responses continued to be detected even during the aviraemic phase when plasma viraemia loads were undetectable in all animals. In general, SIV Gag-specific responses were more robust and consistent than the HIV-1 Env-specific responses in the majority of animals (data not shown). Peak magnitudes of responses varied widely and were measured at different time points p.i. among the animals. The trends of response appear to be similar among the various groups. Nevertheless, the slow increase in antiviral T cell responses suggests subclinical replication of the virus even if plasma viraemia was below the detection levels of our qRT-PCR assay.

### 3.7. Virus Neutralization Assay

**Figure 4:**
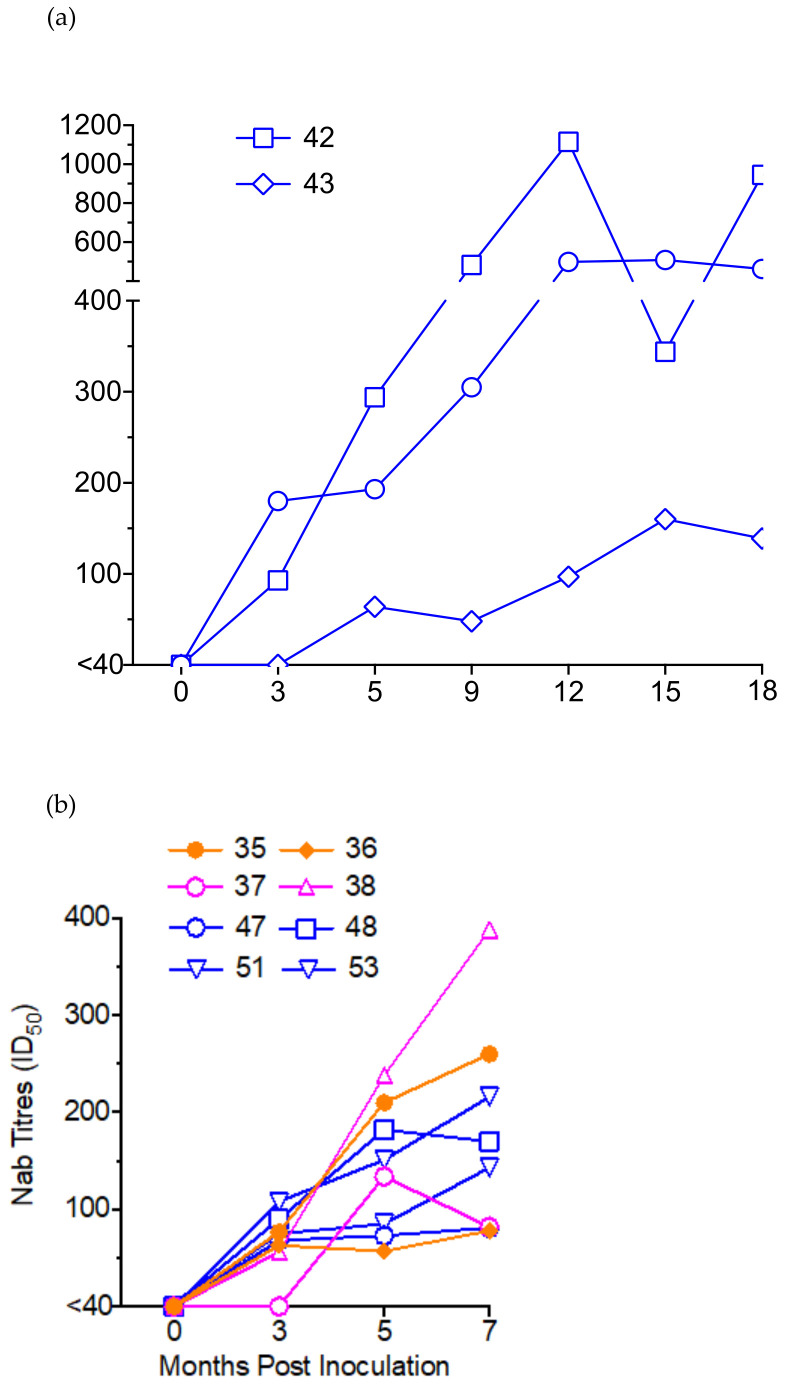
Neutralizing antibody titres to Tier 1A pseudovirus MW965.26 elicited in ChRMs following infection. (**a**) ChRMs inoculated IV with the passaged viruses. (**b**) ChRMs inoculated IR with 1:1 dilution (35 and 36), 1:10 dilution (37 and 38) and 1:100 dilution (47, 48, 51 and 53) of SHIVC109P7. The line graphs represent the antibody titre that caused a 50% reduction in relative luciferase units. A titre <40 was considered negative for neutralization.

Sera from all SHIV-infected ChRMs, both IV- and IR- infected were evaluated for neutralizing activity against clade C, Tier 1A pseudovirus MW965.26 and 12 strains of a Tier 2 global panel of Env-pseudotyped viruses at pre-inoculation and various time points p.i., up to 18 months p.i. in IV-infected animals. The majority of the animals (9 of 11) developed neutralizing antibody (Nab) responses of variable magnitudes against Tier 1 strain MW965 by three months p.i. and all by 5 months and subsequent time points p.i. (Figure 4). At 5 months p.i. the magnitudes of the titres were similar in both IV- and IR- infected animals with median titres at 193 (range: 64–294) and 142.5 (range: 57–238) respectively. Nab titres increased steadily over time, reaching a peak at 12–15 months p.i. in IV-infected animals. No neutralization was detected against the pseudovirions in the Tier 2 global panel.

### 3.8. Full-Length Envelope Sequencing

**Figure 5:**
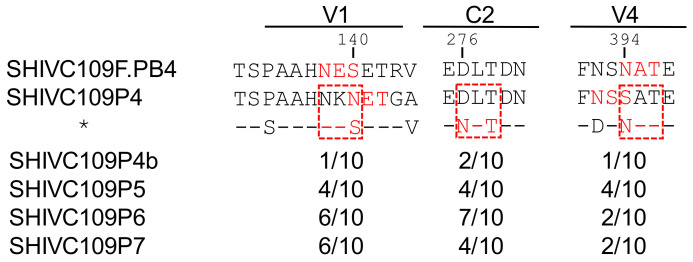
Amino acid sequence comparison of potential N-glycosylation sites (PNGS) in the V1, C2 and V4 regions of SHIVC109F.PB4, SHIVC109P4 and viruses isolated following serial passage of SHIVC109P4 in ChRMs. HIV-1 *env* single genome amplicons (10 per sample) were prepared from SHIVC109P4b (virus inoculum generated from in vitro expansion of SHIVC109P4 in ChRM PBMC), SHIVC109P5, SHIVC109P6 and SHIVC109P7 (virus from passage 1, 2 and 3 respectively). The numbers of amplicons matching the indicated sequence (*) is shown for the inoculum and each passage (per 10 clones sequenced). PNGS are shown in red font and new PNGS are indicated with red boxes.

Sequence analysis of clones isolated prior to passaging in Chinese origin rhesus macaques and after each passage showed that changes to potential N-linked glycosylation sites (PNGS) in the variable region 1 (V1), glycan N276 in the conserved region 2 (C2) and variable region 4 (V4) had occurred in some of the clones (Figure 5 and Supplementary Figure S2). The changes in the V1 and V4 regions resulted in repositioning of the PNGS to those seen in the original SHIVC109F.PB4 virus [18], whereas the change in the C2 region resulted in the creation of an additional PNGS. Interestingly, this was seen in seven out of the ten clones sequenced from the passage two viruses but dropped to only four out of the ten clones in the passage three viruses. None of the changes resulted in the loss of PNGS. Overall, passaging in ChRMs resulted in very few changes to the envelope sequence as shown by the high amino acid sequence identity of >98% between the parental virus (SHIVC109P4) prior to passage and our inoculum stocks (SHIVC109P4b and SHIVC109P7 which was generated from the final passage (P3)).

## 4. Discussion

Research efforts in South Africa have resulted in the development of a number of candidate HIV-1 vaccines [32,39,40,41,42,43,44,45,46,47,48,49,50,51,52,53] including SAAVI DNA-C2 and SAAVI MVA-C, the only two African-produced candidates ever to have successfully moved from basic research to Phase 1 clinical trials [54,55]. These efforts have also led to the establishment of the Chacma baboon and ChRM as nonhuman primate models for the immunogenicity evaluation of these vaccine candidates [32,39,40,43,44,45,56]. In the current study, we sought to enhance this local research capacity by establishing a virus challenge protocol using our locally-available ChRM model, to enable us to conduct future preclinical evaluations of vaccine efficacy in South Africa as well as other HIV prevention and cure strategies. We report here the successful mucosal infection of ChRMs with serially passaged SHIVC109P3, a CCR5-utilizing SHIV containing Env sequences derived from a southern African subtype C HIV-1 and the generation of a suitable ChRM-adapted SHIV inoculum which was titrated for the rectal route, leading to the determination of a dose of SHIVC109P3 suitable for repeated low-dose intra-rectal challenges for future HIV vaccine efficacy studies.

We first characterized the virus stocks generated from the seeding stock and the virus from the P3 passage animal. We confirmed that, our SHIVC109P7-generated virus stock maintained the exclusive utilization of the CCR5 co-receptor (Figure 1c,d) as previously reported [18]. We further confirmed this using human cell lines and showed that both our SHIVC109P4-generated stocks, SHIVC109P4b and SHIVC109P7, replicated in vitro to detectable levels only cell lines that express R5 but not solely X4 co-receptors (Figure 1b). Although the intent for the three rapid in vivo passages of SHIVC109P4 was to adapt the virus to ChRMs, these passages did not show evidence of enhanced replication characteristics in vivo, based on comparable peak viral RNA loads and the rapid and durable control of virus replication by the monkeys (Figure 2a). Sequencing of the virus pre and post in vivo passages nevertheless revealed some adaptation in the form of altered potential glycosylation sites, though overall alterations were modest. The low-level viral kinetics and lack of persistent in vivo viraemia contrasts with those reported by Ren et al [18] for the InRMs infected with the parental virus, SHIVC109P4. However, for these studies, animals were depleted for CD8^+^ cells concurrent with virus inoculations, which markedly delays the initiation of antiviral immune responses [57,58]. Viral loads in these InRMs reached a peak of approximately 10^7^ copies/mL and remained at around 10^4^ copies/ml for 60 weeks post inoculation. ChRMs are reported to display lower viral loads and a prolonged progression to disease [5,59], more similar to HIV infection in humans. In the current study, no clinical signs of AIDS-related disease were observed during the 36 weeks of observation. The continued presence and expansion of both cell mediated (Figure 3) and humoral (Figure 4) antiviral responses noted in our ChRMs strongly suggest continued viral replication in vivo albeit at levels too low to lead to detectable plasma viral RNA levels but sufficient to maintain and enhance potent immune responses controlling the persistent virus. Indeed, viral RNA and proviral DNA were detected in the spleen and lymph nodes obtained at necropsy (at up to 3 years after inoculation) confirming the existence of latent reservoirs in these animals. In previous studies, three major histocompatibility complex (MHC) class 1 alleles (Mamu-A*01, -B*08 and -B*17) have been shown to be efficient in controlling the in vivo replication of SIVmac239 in Indian RMs [60,61,62,63,64,65]. In contrast, no specific alleles have been identified in association with low SIV replication in the ChRMs although a few investigators have reported low frequencies and an inconclusive role of these alleles in this subspecies [59,66].

Indeed, while the cumulative SHIV-specific T cell responses measured with IFN-γ T cell ELISpot against the SIV_mac239_ and HIV-1 subtype C Env peptides were of modest magnitudes, typically <200 SFU/million PBMC and which tended to increase with time in all IR infected monkeys (Figure 3). This contrasts with SHIV and SIV infected animals submitted to extended anti-retroviral therapy in which such responses markedly decrease over time [67,68]. Nab titres against Tier 1 pseudovirions too increased steadily over time, reaching a titre of >500 in P1 and P3 animals by 12 months p.i. (Figure 4a) even though these animals became aviraemic by 3 months p.i. This implies the existence of a reservoir of residual virus replicating, stimulating the immune responses but insufficient in magnitude to be detected in blood.

Amino acid changes in the Env glycoproteins have been reported to result in increased replication and ability to deplete CD4 cells [69,70]. Our finding that, despite successful serial in vivo animal-animal passage, the later-passage viruses did not achieve higher replication capacity suggested that little, if any, evolution occurred in the *env* gene during the viral passages. Sequence determinations of the original virus clone and those isolated from the animals used for passages confirmed that only modest changes in the envelope sequence were seen between the viruses isolated prior to (SHIVC109P4b) and those isolated after three in vivo passages (SHIVC109P7). Most of the passaged viruses did not appear to gain or lose any PNGS. However, shifts in the pattern of glycosylation were noted in the V1 and V4 regions in some clones back to that of the original SHIV109F.PB4 clone. In 4 out of 10 passage 3 and 7 out 10 passage 2 clones sequenced, a glycosylation site was gained at position N276 within loop D of the C2 region (Figure 5 and Supplementary Figure S2). A glycan at position N276 causes steric hindrance and hampers the binding of the variable heavy gene-restricted class of broadly neutralizing antibodies (bNabs) targeting the CD4 binding site such as VRC01 [71]. Thus, acquisition of a glycan residue at this position could be a mechanism of escape from these types of CD4bs antibodies rendering the virus more resistant to neutralization. On the other hand, another class of CD4bs bNabs, which include CAP257-RH1 and HJ16, are critically dependent on the N276 glycan so loss of a glycan at this position would make the virus more resistant to this group of bNabs [71,72]. However, binding of these antibodies was found to be incompatible with glycosylated V5 loops and all viral sequences (SHIVC109P4b, SHIVC109P5, SHIVC109P6 and SHIVC109P7) contained V5 loops with three to four PNGS, rendering it unlikely for antibodies to have caused the changes in glycosylation at this site, since antibody responses were only starting at 2 weeks p.i., the time of clone isolations.

Next, the in vivo titration of the SHIVC109P7 by intrarectal challenges lead to the determination that a 1:100 dilution (~6000 TCID_50_) was enough to infect the ChRMs after 2–3 intrarectal challenges (Figure S2), which we therefore consider equivalent to 1 animal infectious dose 50 (AID_50_). Ren et al [18] reported infection of InRMs after a single IR inoculation with the parental virus at 5000 TCID_50_, suggesting more resistance mucosal transmission in ChRM which agrees with previous reports that showed ChRMs to be more resistant to SIV infection than InRMs (9;11). Like the passage animals, our IR-infected animals exhibited transient viraemia peaking at 1–2 weeks p.i. and declining quickly to undetectable levels by 4–9 weeks p.i. However, proviral SHIV was detectable throughout the viraemic and aviraemic stages indicating that the animals were persistently infected. Moreover, both T cell-mediated and Nab responses in the SHIV-infected animals increased steadily over time (Figure 4) despite the decline of viraemia to undetectable levels (Figure 2) in both the IV- and IR-infected animals.

## 5. Conclusions

In conclusion, this study succeeded in generating a ChRM-adapted SHIV inoculum through in vivo serial passages that was titrated in vivo to determine a dose suitable for use in repeated low-dose intrarectal challenges in future HIV vaccine efficacy studies. Overall, this study demonstrated the feasibility of developing local research capacity to conduct preclinical evaluations of vaccine efficacy and the potential of accelerating HIV vaccine development in South Africa. While this study found that infection of locally available ChRMs with SHIVC109P7 resulted in limited in vivo replication, the infection persists and the model is nevertheless suited to address the prevention of mucosal virus acquisition in vaccine or microbicide testing experiments. This finding also warrants additional studies to improve the viral fitness of subtype C-derived SHIVs for ChRM to serve future research in HIV cure and eradication studies, including antiretroviral studies using this model. SHIVs with better viral fitness are likely to induce more persistent viraemia in our ChRM model to make it more useful for such studies.

## Data Availability

Data is contained within the article or Supplementary Material.

## Supplementary Materials

The following are available online at https://www.mdpi.com/1999-4915/13/3/397/s1, Figure S1: Comparison of different co-culture approaches for isolation of SHIV from the PBMC of IV inoculated animals, Figure S2: Amino acid alignment of gp120 sequences from SHIV clones isolated after passaging in ChRMs.
