# Supplementary material for: Infection of Chinese Rhesus Monkeys with a Subtype C SHIV Resulted in Attenuated In Vivo Viral Replication Despite Successful Animal-to-Animal Serial Passages
Source: Viruses. 2021 Mar 2;13(3):397. doi: 10.3390/v13030397 (PMC7998229; doi:10.3390/v13030397)
Supplement: Supplementary file 1 [file viruses-13-00397-s001.pdf]

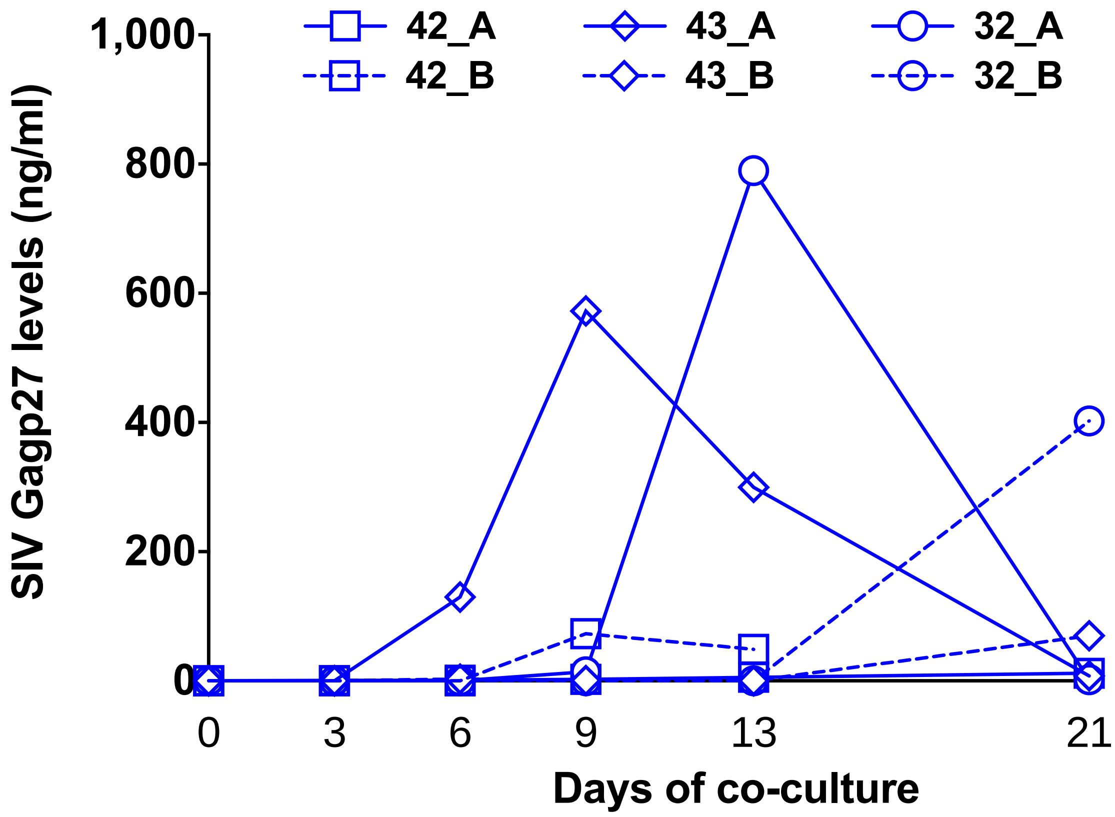

Figure S1: Comparison of different co-culture approaches for isolation of SHIV from the PBMC of IV inoculated animals. Measurement of viral replication in PBMC cultures was set up using two different co-culture approaches as explained in *Materials and Methods.* SIVp27 ELISA was used to determine the level of viral replication. The line graphs show the concentration of the SIVp27 antigen in the co-culture supernatants that were harvested at various time points. A - Co-cultivation of unstimulated PBMC from infected animals. B - Co-cultivation with Con-A-stimulated PBMC from infected animals.

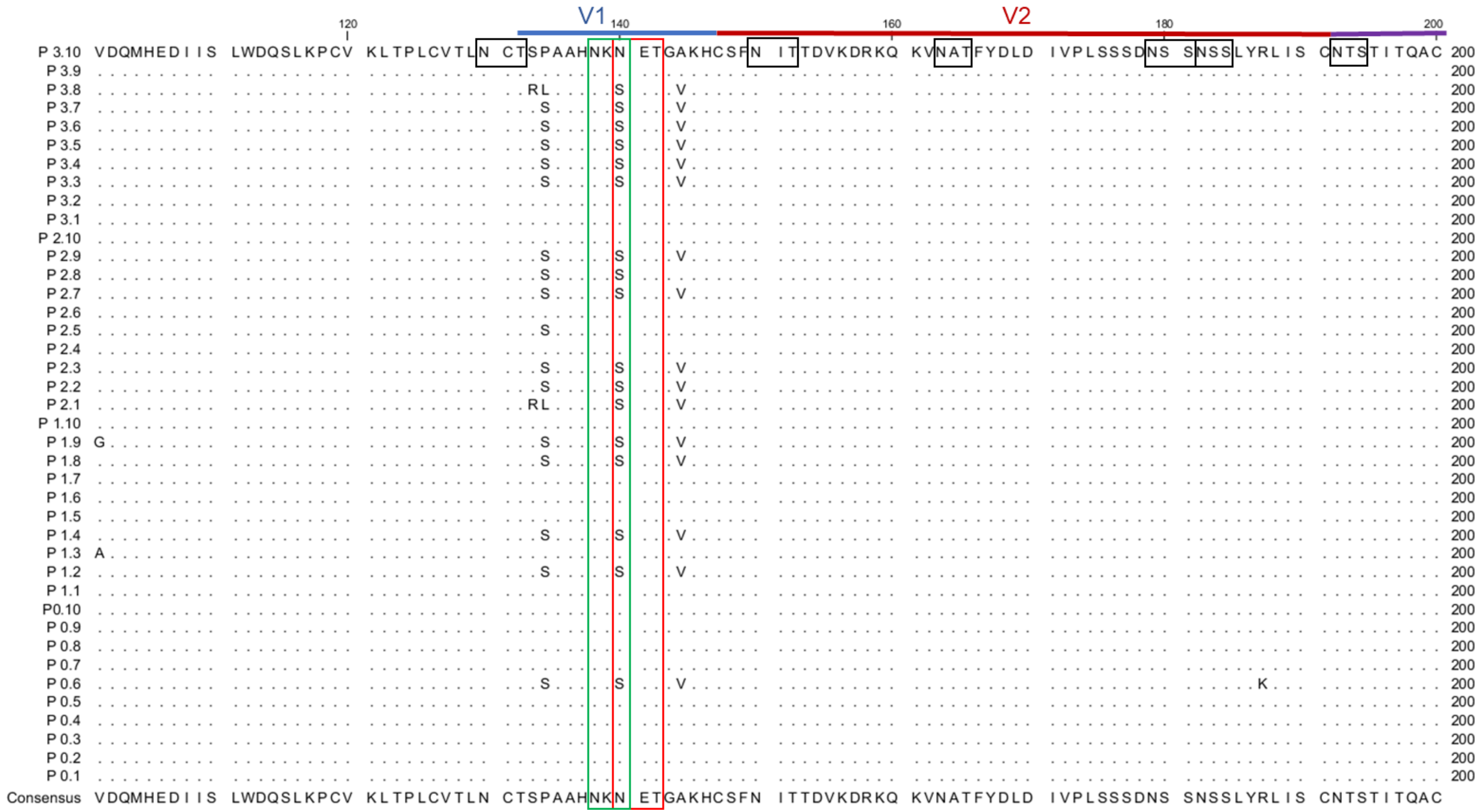
V1
V2
120
140
160
180
200
P 3.10 VDQMHEDIIS LWDQSLKPCV KLTPLCVTLN CTSPAAHNKN ETGAKHCSFN ITTDVKDRKQ KVNATFYDLD IVPLSSSDNS SNSSLYRLIS CNTSTITQAC 200
P 3.9
P 3.8 RL S V
P 3.7 S S V
P 3.6 S S V
P 3.5 S S V
P 3.4 S S V
P 3.3 S S V
P 3.2
P 3.1
P 2.10
P 2.9 S S V
P 2.8 S S
P 2.7 S S V
P 2.6
P 2.5 S
P 2.4
P 2.3 S S V
P 2.2 S S V
P 2.1 RL S V
P 1.10
P 1.9 G S S V
P 1.8 S S V
P 1.7
P 1.6
P 1.5
P 1.4 S S V
P 1.3 A
P 1.2 S S V
P 1.1
P0.10
P 0.9
P 0.8
P 0.7
P 0.6 S S V K
P 0.5
P 0.4
P 0.3
P 0.2
P 0.1
Consensus VDQMHEDIIS LWDQSLKPCV KLTPLCVTLN CTSPAAHNKN ETGAKHCSFN ITTDVKDRKQ KVNATFYDLD IVPLSSSDNS SNSSLYRLIS CNTSTITQAC

C2

```
                    220        240        260        280        300
P 3.10    PKVSFDPIPI HYCAPAGYAI LKCNNKTFSG KGPCSNVSTV QCTHGIRPVV STQLLLNGSL AEEEIVIRSE DLTDNVKTII VHLNKSVEIE CIRPGNNTRR 300
P 3.9     .......... .......... .......... .......... .......... .......... .......... .......... .......... .......... 300
P 3.8     .......... .......... .......... .......... .......... .......... .......... .......... .......... .......... 300
P 3.7     .......... .......... .......... .......... .......... .......... .......... N........M .......... ...T...... 300
P 3.6     .......... .......... .......... .......... .......... .......... .......... .......... .......... .......... 300
P 3.5     .......... .......... .......... .......... .......... .......... .......... N......... .......... ...T...... 300
P 3.4     .......... .......... .......... .......... .......... .......... .......... .......... .......... .......... 300
P 3.3     .......... .......... .......... .......... .......... .......... .......... .......... .......... .......... 300
P 3.2     .......... .......... .......... .......... .......... .......... .......... N......... .......... .......... 300
P 3.1     .......... .......... .......... .......... .......... .......... .......... N......... .......... .......... 300
P 2.10    .......... .......... .......... .......... .......... .......... .......... N......... .......... .......... 300
P 2.9     .......... .......... .......... .......... .......... .......... .......... N......... .......... .......... 300
P 2.8     .......... .......... .......... .......... .......... .......... .......... .......... .......... .......... 300
P 2.7     .......... .......... .......... .......... .......... .......... .......... N......... .......... ...T...... 300
P 2.6     .......... .......... .......... .......... .......... .......... .......... .......... .......... .......... 300
P 2.5     .......... .......... .......... .......... .......... .......... .......... N......... .......... .......... 300
P 2.4     .......... .......... .......... .......... .......... .......... .......... N......... .......... .......... 300
P 2.3     .......... .......... .......... .......... .......... .......... .......... N......... .......... ...T...... 300
P 2.2     .......... .......... .......... .......... .......... .......... .......... N......... .......... .......... 300
P 2.1     .......... .......... .......... .......... .......... .......... .......... .......... .......... .......... 300
P 1.10    .......... .......... .......... .......... .......... .......... .......... .......... .......... .......... 300
P 1.9     .......... .......... ........R. .......... .......... .......... .......... N......... .......... ...T...... 300
P 1.8     .......... .......... ........R. .......... .......... .......... .......... N......... .......... ...T...... 300
P 1.7     .......... .......... .......... .......... .......... .......... .......... .......... .......... .......... 300
P 1.6     .......... .......... .......... .......... .......... .......... .......... .......... .......... .......... 300
P 1.5     .......... .......... .......... .......... .......... .......... .......... .......... .......... .......... 300
P 1.4     .......... .......... .......... .......... .......... .......... .......... N......... .......... .......... 300
P 1.3     .......... .......... .......... .......... .......... .......... .......... .......... .......... .......... 300
P 1.2     .......... .......... .......... .......... .......... .......... .......... N......... .......... .......... 300
P 1.1     .......... .......... .......... .......... .......... .......... .......... .......... .......... .......... 300
P0.10     .......... .......... .......... .......... .......... .......... .......... .......... .......... .......... 300
P 0.9     .......... .......... .......... .......... .......... .......... .......... .......... .......... .......... 300
P 0.8     .......... .......... .......... .......... .......... .......... .......... N......... .......... ...T...... 300
P 0.7     .......... .......... .......... .......... .......... .......... .......... .......... .......... .......... 300
P 0.6     .......... .......... .......... .......... .......... .......... .......... .......... .......... .......... 300
P 0.5     .......... .......... .......... .......... .......... .......... .......... .......... .......... .......... 300
P 0.4     .......... .......... .......... .......... .......... .......... .......... .......... .......... .......... 300
P 0.3     .......... .......... .......... .......... .......... .......... .......... N.A....... .......... ....S....K 300
P 0.2     .......... .......... .......... .......... .......... .......... .......... .......... .......... .......... 300
P 0.1     .......... .......... .......... .......... .......... .......... .......... .......... .......... .......... 300
Consensus PKVSFDPIPI HYCAPAGYAI LKCNNKTFSG KGPCSNVSTV QCTHGIRPVV STQLLLNGSL AEEEIVIRSE DLTDNVKTII VHLNKSVEIE CIRPGNNTRR
```

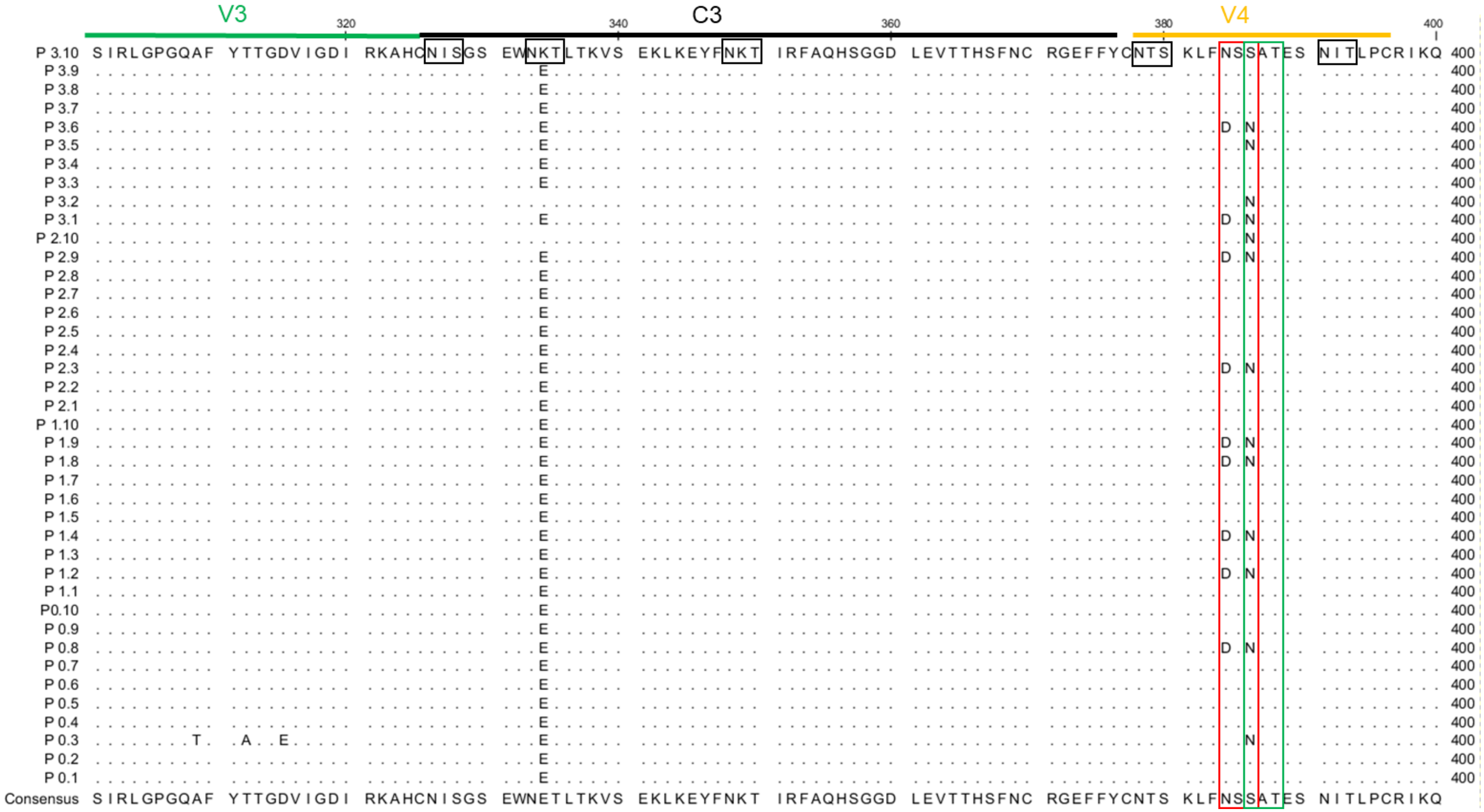

Figure S2. Amino acid alignment of gp120 sequences from SHIV clones isolated after passaging in ChRMs. P0 = virus inoculum prepared in rhesus macaque PMBCs. Virus isolated 2 weeks post infection from the first passage = P1, second passage = P2 and third passage = P3. Potential N-glycosylation sites (PNGS) are bracketed with black boxes,

red boxes indicate where glycosylation sites have been lost and green boxes sites where new sites have been gained. Variable regions (V) and conserved regions (C) are shown above the sequences.
